# Expanding the Nude SCID/CID Phenotype Associated with FOXN1 Homozygous, Compound Heterozygous, or Heterozygous Mutations

**DOI:** 10.1007/s10875-021-00967-y

**Published:** 2021-01-19

**Authors:** Giuliana Giardino, Svetlana O. Sharapova, Peter Ciznar, Fatima Dhalla, Luca Maragliano, Akella Radha Rama Devi, Candan Islamoglu, Aydan Ikinciogullari, Sule Haskologlu, Figen Dogu, Rima Hanna-Wakim, Ghassan Dbaibo, Janet Chou, Emilia Cirillo, Carla Borzacchiello, Alexandra Y. Kreins, Austen Worth, Ioanna A. Rota, José G. Marques, Muge Sayitoglu, Sinem Firtina, Moaffaq Mahdi, Raif Geha, Bénédicte Neven, Ana E. Sousa, Fabio Benfenati, Georg A. Hollander, E. Graham Davies, Claudio Pignata

**Affiliations:** 1 Department of Translational Medical Sciences, Pediatrics Section, Federico II University of Naples, via S. Pansini 5, 80131 Naples, Italy; 2 Research Department, Belarusian Research Center for Pediatric Oncology, Hematology and Immunology, Minsk, Belarus; 3 Pediatric Department, Faculty of Medicine, Comenius University in Bratislava, National Institute of Childhood Diseases, Bratislava, Slovakia; 4 Department of Paediatrics and the Weatherall Institute of Molecular Medicine, University of Oxford, Oxford, UK; 5 Center for Synaptic Neuroscience and Technology, Istituto Italiano di Tecnologia, 16132 Genova, Italy; 6 IRCCS Ospedale Policlinico San Martino, 16132 Genova, Italy; 7 Sandor Life Sciences Pvt. Ltd., Banjara Hills, Hyderabad, India; 8 Department of Pediatric Immunology and Allergy, Ankara University Medical School, Ankara, Turkey; 9 Department of Pediatrics and Adolescent Medicine, American University of Beirut, Beirut, Lebanon; 10 Division of Immunology, Boston Children’s Hospital and Department of Pediatrics, Harvard Medical School, Boston, MA USA; 11 Department of Paediatric Immunology, Great Ormond Street Hospital and UCL Great Ormond Street Institute of Child Health, London, UK; 12 Instituto de Medicina Molecular, Faculdade de Medicina, Universidade de Lisboa, Lisboa, Portugal; 13 Unidade de Infecciologia e Imunodeficiências, Departamento de Pediatria, Centro Hospitalar Universitário Lisboa Norte, Lisboa, Portugal; 14 Department of Genetics, Aziz Sancar Institute of Experimental Medicine, Istanbul University, Istanbul, Turkey; 15 Faculty of Art and Science, Istinye University, Istanbul, Turkey; 16 King Saud bin Abdulaziz University for Health Sciences College of Medicine, Jeddah, Saudi Arabia; 17 Paediatric Haematology-Immunology and Rheumatology Department, Hôpital Necker-Enfants Malades, Assistance Publique-Hôpitaux de Paris (AP-HP), Université de Paris, Institut IMAGINE, Paris, France; 18 Paediatric Immunology, Department of Biomedicine, University of Basel, The Basel University Children’s Hospital, 4052 Basel, Switzerland

**Keywords:** Nude SCID, FOXN1, homozygous, compound heterozygous, heterozygous, Omenn syndrome, alopecia, nail dystrophy, EBV-related lymphoproliferative disease

## Abstract

Human nude SCID is a rare autosomal recessive inborn error of immunity (IEI) characterized by congenital athymia, alopecia, and nail dystrophy. Few cases have been reported to date. However, the recent introduction of newborn screening for IEIs and high-throughput sequencing has led to the identification of novel and atypical cases. Moreover, immunological alterations have been recently described in patients carrying heterozygous mutations. The aim of this paper is to describe the extended phenotype associated with *FOXN1* homozygous, compound heterozygous, or heterozygous mutations. We collected clinical and laboratory information of a cohort of 11 homozygous, 2 compound heterozygous, and 5 heterozygous patients with recurrent severe infections. All, except one heterozygous patient, had signs of CID or SCID. Nail dystrophy and alopecia, that represent the hallmarks of the syndrome, were not always present, while almost 50% of the patients developed Omenn syndrome. One patient with hypomorphic compound heterozygous mutations had a late-onset atypical phenotype. A SCID-like phenotype was observed in 4 heterozygous patients coming from the same family. A spectrum of clinical manifestations may be associated with different mutations. The severity of the clinical phenotype likely depends on the amount of residual activity of the gene product, as previously observed for other SCID-related genes. The severity of the manifestations in this heterozygous family may suggest a mechanism of negative dominance of the specific mutation or the presence of additional mutations in noncoding regions.

## Introduction

Nude severe combined immunodeficiency (SCID) syndrome is an autosomal recessive disorder characterized by congenital alopecia, nail dystrophy, and athymia. It was described for the first time in 1996 as the human equivalent of the well-known nude murine phenotype [1]. The phenotype arises from the biallelic loss of function mutations of the *FOXN1* gene, a member of the forkhead box gene family that includes a diverse group of “winged helix” transcription factors implicated in a variety of cellular processes: development, metabolism, cancer, and aging [2–4]. During postnatal life, *FOXN1* is expressed in thymic stromal and skin cells, where it is necessary for the normal development, function, and maintenance of hair follicles and thymic epithelial cells (TECs) [5–7]. After the description of the first two cases, only few patients have been described in almost 15 years [3, 8–12]. However, during the last few years, newborn screening (NBS) and next-generation sequencing (NGS) led to the identification of an increased number of patients, suggesting that the actual incidence of the disease is higher than previously thought and many patients die before receiving a diagnosis [13–15]. In contrast to most the other forms of SCID, nude SCID is not related to a defect in the hematopoietic stem cells (HSC) but to a defect of the thymic epithelial stroma and this makes the definitive treatment of these conditions more complicated [16].

As for other inborn errors of immunity (IEIs), NGS allowed the diagnosis also in patients presenting with atypical phenotypes, broadening the spectrum of clinical manifestations related to different *FOXN1* mutations [17, 18]. In a recent study, we described a cohort of subjects found positive on NBS in which NGS revealed heterozygous mutations in the *FOXN1* gene [17]. All the pediatric patients showed lymphopenia with a tendency to resolution in older ages and about half had recurrent upper respiratory tract infections. In a few cases, the phenotype was more severe, characterized by chronic or severe invasive opportunistic or viral infections, suggesting a more severe dysfunction of FOXN1.

In this context, the aim of this international survey is to define the spectrum of clinical and laboratory phenotypes associated with *FOXN1* heterozygous, homozygous, or compound heterozygous mutations, and to describe novel clinical findings associated with FOXN1 deficiency.

## Methods

### Patients

We enrolled a cohort of 18 patients from 11 families in a worldwide collaboration. The study was approved by the institutional Ethical committee “Carlo Romano” of Federico II University. Before inclusion in the study, all the patients underwent genetic evaluation by linkage analysis, Sanger sequencing, or NGS. Patients included in the study carried homozygous or compound heterozygous mutations of the *FOXN1* gene. We also included 5 patients, coming from the same family, who showed symptoms suggestive of combined immunodeficiency, despite carrying a heterozygous mutation. Most of the patients have been previously described [1, 3, 8–15, 17]. Informed consent was obtained from the patients or from their parents for inclusion in the study.

Clinical and laboratory data were retrieved retrospectively from the clinical records. Demographics, age at diagnosis, and information on clinical manifestations at diagnosis and during follow-up were collected. In particular, we focused our attention on the presence of nail dystrophy, alopecia, and susceptibility to infections, autoimmunity, and malignancies. Moreover, information on treatment and outcome was also collected. Laboratory information included absolute lymphocyte count, lymphocyte subpopulations including CD3+, CD4+, CD8+, naïve CD4+CD45RA+, CD8+CD45RA+ T cells, CD4+CD31+CD45RA+ (recent thymic emigrants, RTE), CD19+ B cells, CD16+56+ NK cells, TCRαβ, TCRγδ, TCRαβ+ CD4-CD8-, IgG, IgA, IgM, proliferative response to PHA and PMA/ionomycin, and TCRVβ repertoire.

### Crystal Structure of Human FOXN1 in Complex with DNA

The crystal structure of human FOXN1 in complex with DNA [19] (PDB code: 6EL8) was used to visualize the sites of mutations in the DNA binding forkhead domain. Mutants were generated using the Chimera software [20]. Protein-DNA binding affinity changes upon mutations were evaluated using two different methods, mCSM [21] and SAMPDI [22]. mCSM shows the structural environment around the mutated residue with graph-based signatures and relies on supervised machine learning to train predictive models for protein-nucleic acid interactions using thermodynamic data sets. SAMPDI integrates knowledge-based terms with the widely adopted molecular mechanics Poisson–Boltzmann surface area (MM/PBSA) [23], which combines molecular mechanic calculations with continuum solvation models.

### Functional Validation of Variants of Unknown Significance

Human WT *FOXN1* cDNA containing a stop codon at the end of its sequence (sequence accession number BC140423) was cloned into the pCSF107mT-GATEWAY-3′-FLAG (Addgene). Insertion of single base pair changes for the generation of the human P350L FOXN1 variant was achieved by site-directed mutagenesis using the Phusion site-directed mutagenesis kit (#F-541, Thermo Fisher Scientific). Primer pairs (F: 5’GTCGATCTTGGCCAGATTGAGG3’ and R: 5’CCTCAATCTGGCCAAGATCGAC3’) for the introduction of the single base pair change found in the patient were designed using the software http://www.genomics.agilent.com/primerDesignProgram.jsp?toggle=uploadNow&mutate=true&_requestid=295758. Correct incorporation of the single base pair change was verified by Sanger sequencing.

The human-derived thymic epithelial cell line, 4D6, was used for the luciferase assay. Cells were seeded in 24-well cell culture plates and grown to 70–80% confluence for transfection using Fugene (Promega) as per manufacturer’s recommendations. A luciferase reporter gene pGL4.10 (Luc2, Promega) was cloned downstream of a wild-type β5t promoter (β5t-luc) or a mutated β5t promoter with a mutated FOXN1 binding site (β5t-mut-luc), with β5t being a known FOXN1 target. Each condition was transfected with a luciferase reporter plasmid (β5t-luc or β5t-mut-luc), a Renilla control plasmid (pRL Promega), and a *FOXN1* construct of interest in a ratio of 10:1:10. Each transfection was performed in triplicate. Twenty-four hours post-transfection, cell lysates were prepared following the manufacturer’s protocol (Promega Dual Luciferase reporter assay system) using 80 μl/well of PLB lysis buffer provided by the kit. Luciferase readings were performed at a Promega Glo Max luminometer. Reporter activity was corrected by calculating the ratio of luciferase/Renilla for each well. The activity of luciferase was reported as relative luciferase units (RLU). Luciferase and Renilla plasmids were kindly provided by Dr. Saulius Zuklys (Department of Biomedicine, University of Basel).

## Results

### Genetics

**Fig. 1:**
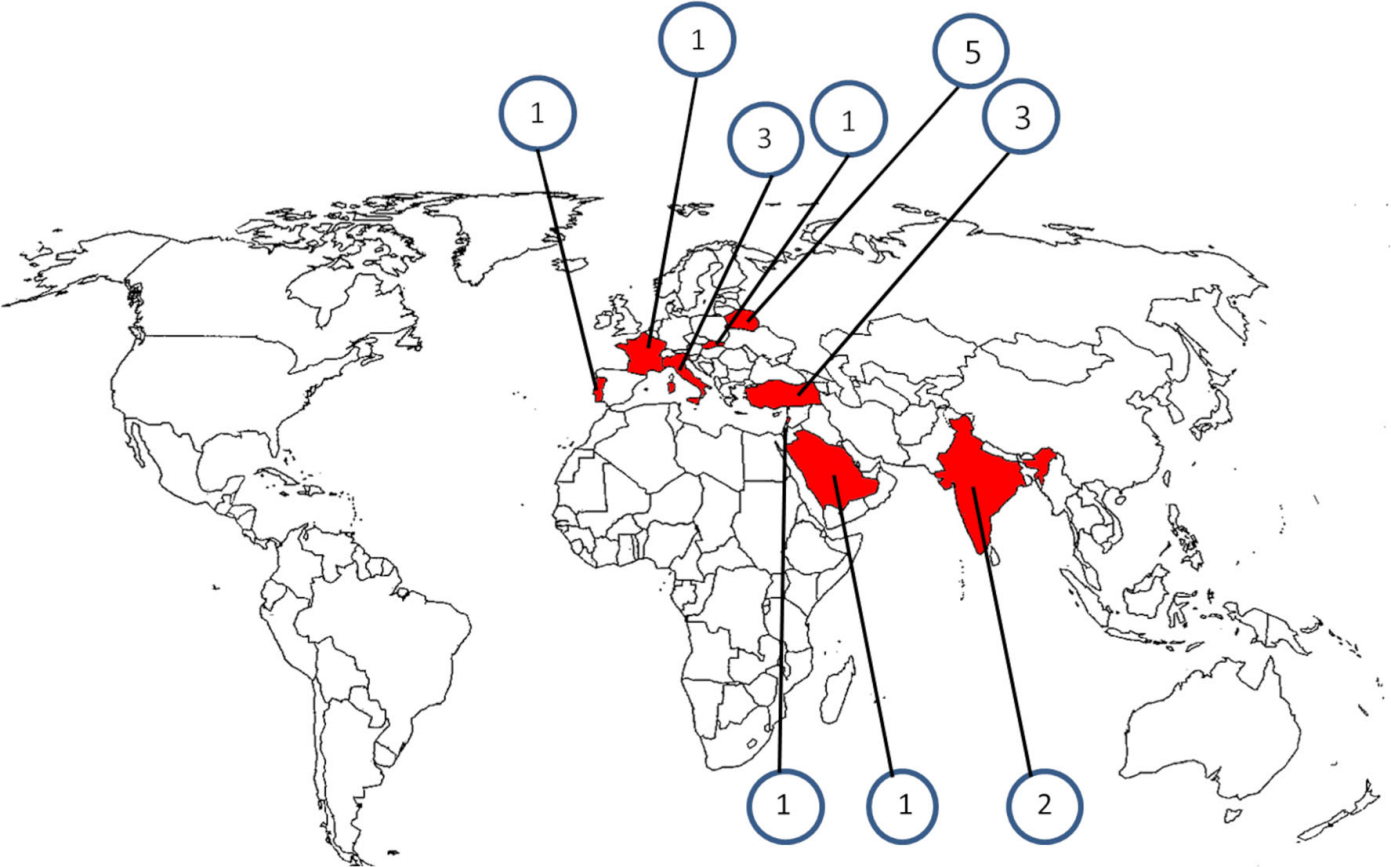
Geographic distribution of the cases included in the study. Patients originated from 9 countries (Italy, Portugal, France, Lebanon, India, Turkey, Saudi Arabia, Slovakia, Belarus)

**Fig. 2:**
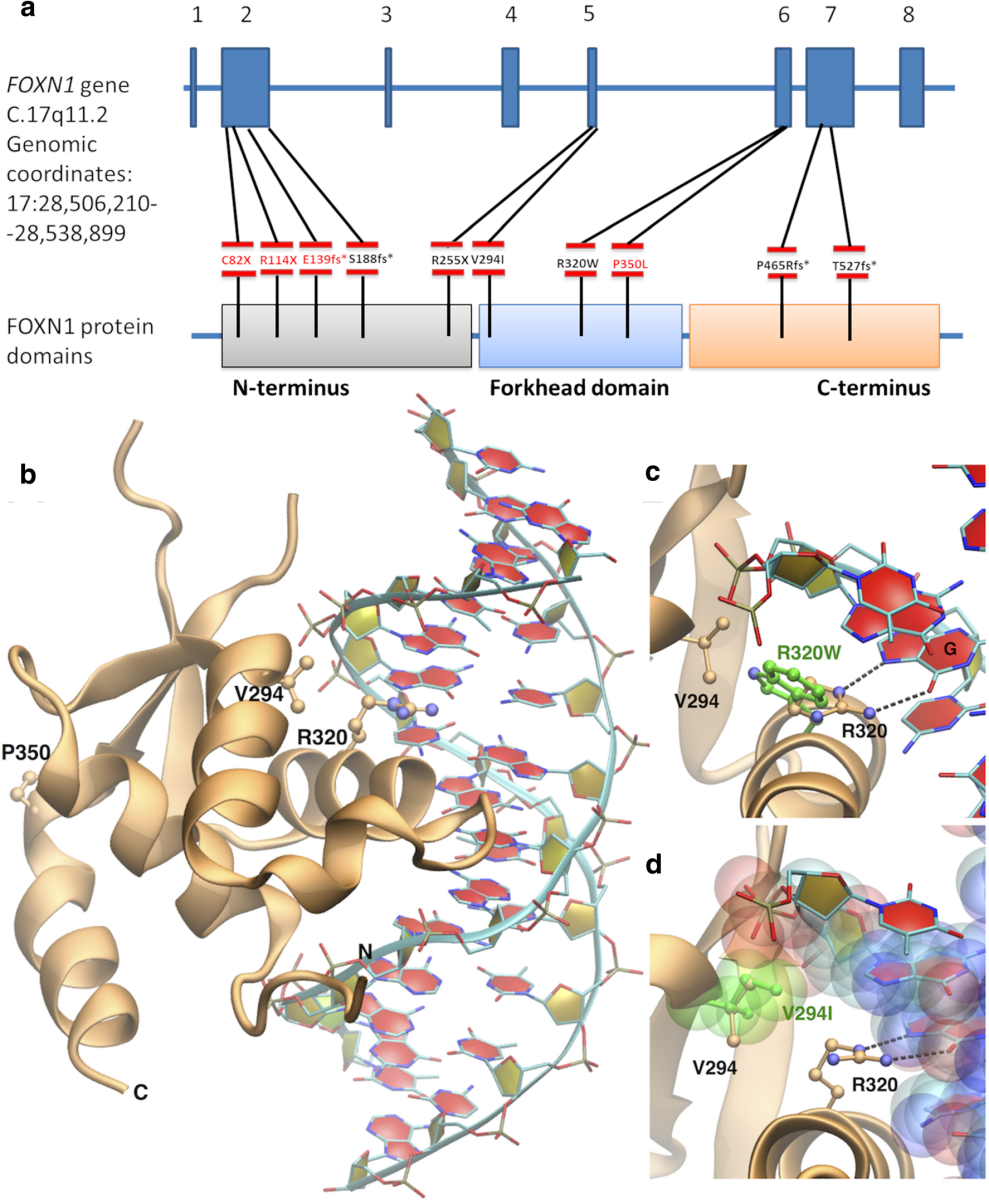
Mutations identified in the patients and crystal structure of FOXN1/DNA complex. **a** Five mutations were identified in the N-terminal domain, three in the forkhead domain, and two in the C-terminal domain. Novel mutations are highlighted in red. **b** Structure of the complex with the protein represented as ribbons. The three mutated residues in the forkhead domain are represented as ball-and-stick. **c** Enlarged view of the protein-DNA interface with the mutated R320W reported in green. **d** Same as in **c** but for V294I, and with protein and DNA atoms represented as transparent spheres of van der Waals radius

Eighteen patients from 11 families originating from 9 countries (Italy, Portugal, France, Lebanon, India, Turkey, Saudi Arabia, Slovakia, Belarus) were included in the study (Fig. 1). The male/female (M/F) ratio was 1:1.25. In total, 11 of the 18 cases (61%) were familial (4 families), and the remaining 7 cases were sporadic. Family history revealed a further female subject (cousin of P5; data not included in the study) who died at the age of 4 months, with very high levels of IgE. Her parents are consanguineous and both heterozygous for the same *FOXN1* mutation. Ten different mutations in *FOXN1* were identified (Fig. 2a). Mutations affecting the N-terminal domain were the most common (50%), followed by those in the forkhead domain (30%), and in the C-terminal domain (20%). In total, we identified 4 novel mutations, namely R114X, E139fs, C82X, and P350L (Fig. 2a). Eleven patients were homozygous for the mutation, 2 patients were compound heterozygous, and 5 were heterozygous. In the 2 patients with compound heterozygous mutations, P8 carried 2 stop mutations while P9 had a stop mutation and a missense mutation. In 8 out of 18 patients, there was a history of consanguinity. Four mutations caused frameshifts leading to the generation of premature stop codons. Three mutations (2 in the forkhead and 1 in the C terminus) were missense.

We used the recently solved crystal structure of human FOXN1 in complex with DNA [19] (PDB code: 6EL8) to map the mutated residues and to quantify the effect of their substitution on protein-DNA binding. The structure comprises protein residues from 269 to 362, belonging to the forkhead domain, and a dsDNA sequence of 13 nucleotides containing the consensus sequence for FOXN1 binding, 5′-GACGC. The complex reveals a specific protein-DNA interface where the DNA sequence is recognized by residues mostly from an α-helix inserted in the DNA major groove, usually called the recognition helix. Three of the mutations presented in this study (V294I, R320W, and P350L) are within the forkhead domain (Fig. 2b). R320 is in the recognition helix and it is part of the DNA recognition pattern, establishing two hydrogen bonds with the guanine of the fifth base pair of the consensus sequence. Substituting R320 with the bulkier, hydrophobic residue tryptophan results in loss of this interaction (Fig. 2c). V294 is also at the protein-DNA interface, with one of its carbon atoms at 4 Å from the dsDNA backbone, and substitution with the longer side chain of isoleucine could create steric hindrance at the interface (Fig. 2d). At variance with the previous two amino acids, P350 is not part of the protein-DNA interface since it is found at the end of the forkhead C-terminal helix. However, proline is known to be a helix breaker, and its substitution with leucine, one of the most helix-stabilizing residues, can be expected to have a major impact on the protein folding essential for DNA binding. We then used two different programs, mCSM [21] and SAMPDI [22], to quantify changes in FOXN1-DNA binding affinity induced by the three mutations in the forkhead domain. Consistent with the structural observations above, both methods predicted destabilizing effects for all three mutations. Specifically, for V294I, R320W, and P350L, mCSM yielded changes in binding free energy of − 0.385 kcal/mol, − 0.617 kcal/mol, and − 0.498 kcal/mol, while SAMPDI gave − 0.218 kcal/mol, − 0.249 kcal/mol, and − 0.555 kcal/mol, respectively, where a negative value indicates a weaker binding in the mutated complex compared to the native one.

### Clinical Manifestations

Clinical manifestations are summarized in Table 1. All the homozygous/compound heterozygous patients presented with signs suggestive of CID, including severe bacterial, viral or fungal infections, diarrhea, and failure to thrive. The mean age at onset was 3.75 months, ranging from 5 days to 18 months.

**Table 1:** Clinical manifestations in patients with FOXN1 homozygous, compound heterozygous and heterozygous mutations presenting with typical SCID phenotype

	P1	P2	P3	P4	P5	P6	P7	P8	P9	P10	P11	P12	P13	P14	P15	P16	P17	P18
Sex	F	F	F	M	F	F	M	F	M	F	M	M	F	F	M	M	F	M
Age at onset (months)	2	2	3	3	1	0.5	NA	0.5	18	3	4	2	6	0.3	1	Childhood	-	0.5
Country of origin	Italy	Italy	Portugal	France/Africa	Lebanon	India	India	Italy	Slovakia	Turkey	Saudi Arabia	Turkey	Turkey	Belarus	Belarus	Belarus	Belarus	Belarus
Protein effect	R255X	R255X	R255X	R320W	S188fs	R255X	R255X	R114X/E139fs	C82X/P350L	R114X	T527fs*	V294I	V294I	P465Rfs*82	P465Rfs*82	P465Rfs*82	P465Rfs*82	P465Rfs*82
Zygosity	Homo	Homo	Homo	Homo	Homo	Homo	Homo	Compound het	Compound het	Homo	Homo	Homo	Homo	Het	Het	Het	Het	Het
Alopecia	+	+	+	+	−/+	+	+	+	+/−	+	+	−	+	+ (eyebrows)	+ (eyebrows)	+ (eyebrows)	−	+ (eyebrows)
Nail dystrophy	+	+	+	+	−	+	+	+	−	+	+	−	+	+	+	+	+	+
Epicanthic folds	+	+	−	−	−	+	−	+	−	−	−	−	+	−	−	−	−	−
Failure to thrive	+	−	−	−	−	−	−	−	−	+	−	−	+	+	+	−	−	−
Chronic diarrhea	+	−	+	-	+	+	+	+	−	+	−	+	−	+	+	−	−	−
Pneumonia	+	+	+	+	−	+	−	+	−	+ (CMV)	−	+	+	+	+	−	−	+
Other infections	−	Pyogenic Infections	*Mycobacterium bovis* pneumonia, BCG adenitis	HHV6	−	Oral candidosis	Oral candidosis	−	Generalized lymphadenopathy, high EBV viral load, EBV-driven infiltrative lung disease	BCG adenitis	Pseudomonas aeruginosa sepsis	Otitis, oral candidosis	Sepsis, oral candidosis	Omphalitis, candidosis CMV infection, molluscum contagiosum, partial atrophy of the optic nerves, bilateral disseminated chorioretinitis	CMV, HHV-6, enterocolitis	Sinusutis, staphylococcal, and *Salmonella* sepsis, hepatitis A and B	−	Omphalitis
Autoimmunity	−	−	−	−	−	−	−	−	+	−	−	−	−	−	−	+	+	−
Omenn syndrome/ erythroderma	+	+	+	−	+	−	−	+	−	+	−	−	+	−	−	−	−	+
EBV-related B cell lymphoma	−	−	−	−	−	−	−	−	+	−	+	−	−	+	−	−	−	−
Treatment	None	HSCT	TT	TT	HSCT	HSCT	None	TT	TT	TT	None	None	HSCT	IVIG	HSCT	None	None	IVIG, Cotrimoxazole and Acyclovir prophylaxis
Outcome	Dead	Alive (22 yo)	Alive (14.5 yo)	Alive (13 yo)	Dead (5 mo)	Dead	Dead	Dead	Alive (12 years)	Alive (3 years and 5 months)	Dead	Dead	Alive	Alive (11 yo)	Dead	Alive (38 yo)	Alive (61 yo)	Alive (4 mo)
Reference	[1]	[1]	[9, 11]	[10, 11]	[12]	[13]	[13]	−	−	−	[15]	[14]	[14]	[17]	[17]	−	−	−

*BCG*, Bacillus Calmette–Guerin; *HHV6*, human herpes Virus 6; *EBV*, Epstein–Barr virus; *CMV*, cytomegalovirus; *HSCT*, hematopoietic stem cell transplant; *TT*, thymus transplant; *IVIG*, intravenous immunoglobulins

Alopecia was observed at the onset in all but two homozygous/compound heterozygous patients (P5 and P12), while nail dystrophy was observed in 10 out of 13 patients. In P5, carrying the S188fs mutation, nail dystrophy was not present, and alopecia only appeared after the development of erythroderma, while P9 showed spontaneous hair regrowth starting from 3 years of age. Seven out of 13 CID/SCID patients (53.8%) showed erythroderma or Omenn syndrome (OS).

Apart from ectodermal alterations, the most common clinical manifestations were diarrhea, failure to thrive, and pneumonia, observed in 11 patients. Lung involvement was characterized by severe bronchopneumonia and interstitial pneumonia in 4 patients (P1, P2, P9, and P10), respiratory failure requiring mechanical ventilation in 2 patients (P3 and P4), and two episodes of pneumonia partially responding to the treatment in one patient (P6). Persistent oral candidosis was observed in 4 patients (P6, P7, P12, and P13). Other clinical manifestations included *Mycobacterium bovis* pneumonia in P3; Bacillus Calmette–Guerin (BCG) adenitis in P3 and P10; human herpesvirus 6 (HHV-6) infection leading to anemia, neutropenia, and thrombocytopenia in P4; and *Pseudomonas aeruginosa* sepsis in P11.

**Fig. 3:**
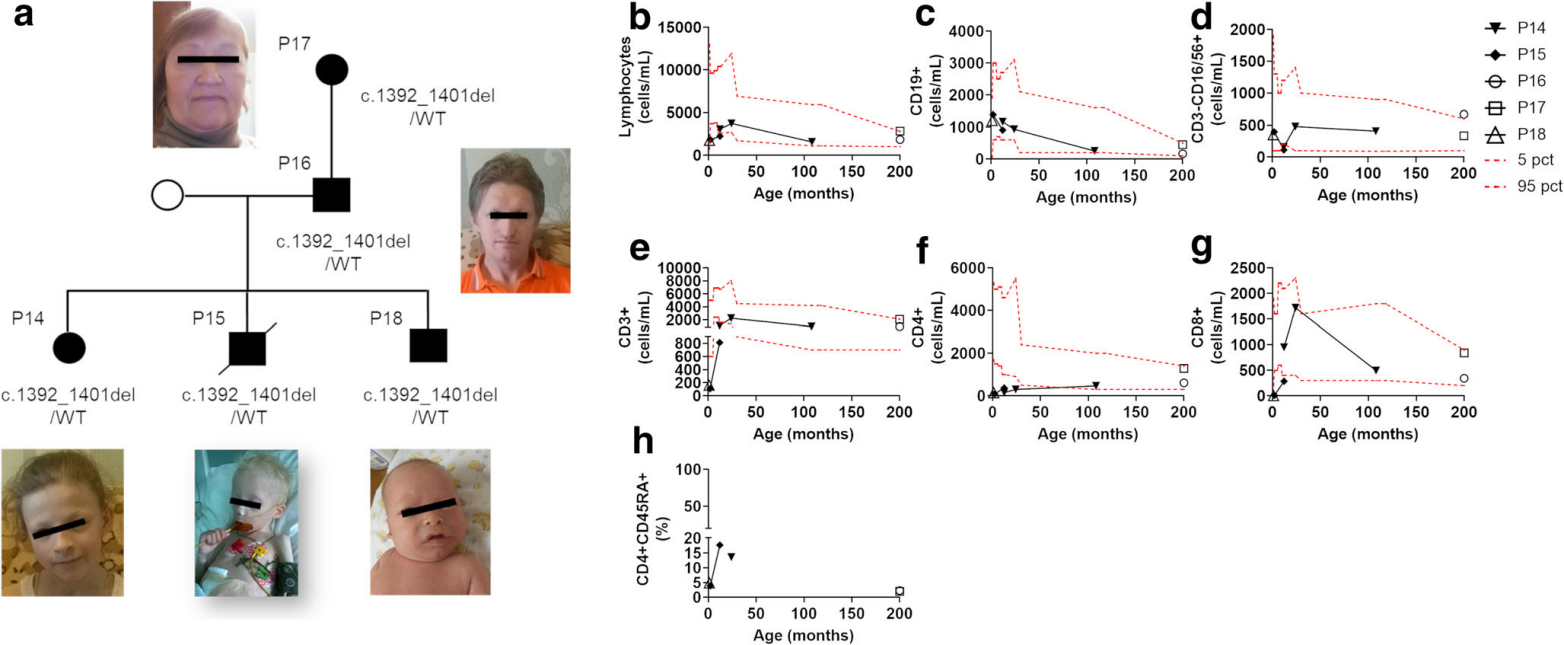
Heterozygous patients’ family pedigree and lymphocyte subpopulations at different ages. **a** Healthy subjects are shown in white; carriers are shown in black; deceased siblings are indicated by line crossing. **b** Lymphocytes. **c** B cells (CD19+). **d** NK cells (CD16/CD56+). **e** T cells (CD3+). **f** T helper (CD3+CD4+). **g** Cytotoxic T cells (CD3+CD8+). **h** Naïve T helper (CD3+CD4+CD45RA+). In all the panels, each line and symbol represents a single patient. The red dashed lines represent the 5th and 95th centile levels of the age-matched reference values

Heterozygous patients included 5 patients coming from a single family (Fig. 3a). *FOXN1* mutation was identified through NGS in the 2 siblings presenting in the first month of life with a CID phenotype characterized by pneumonia, diarrhea, and failure to thrive. The Invitae Primary Immunodeficiency Panel, including 207 immunity genes, was used to investigate the patients. Moreover, they also suffered from other infections, including omphalitis, CMV infection, molluscum contagiosum, bilateral disseminated chorioretinitis with partial atrophy of the optic nerves in P14, and CMV and HHV-6 infections in P15. The same heterozygous mutation was also identified prenatally in the third sibling who developed erythroderma, pneumonia, and omphalitis in the first weeks of life (P18). The father (P16) and the grandmother (P17) also carried the mutation. P16 developed severe infections during childhood, including *Staphylococcus* and *Salmonella* sepsis which required admission to the intensive care unit on three different occasions. Susceptibility to infections in P16 improved dramatically during follow-up. No history of recurrent or severe infections was reported in P17. Alopecia was observed in 4/5 heterozygous patients (P14, P15, P16, and P18), but it was limited to the eyebrows, while nail dystrophy was observed in all the heterozygous patients.

None of the patients suffered from neurological disorders and a history of recurrent abortions was reported by the mother of P6 and P7.

### Atypical Clinical Phenotypes

**Fig. 4:**
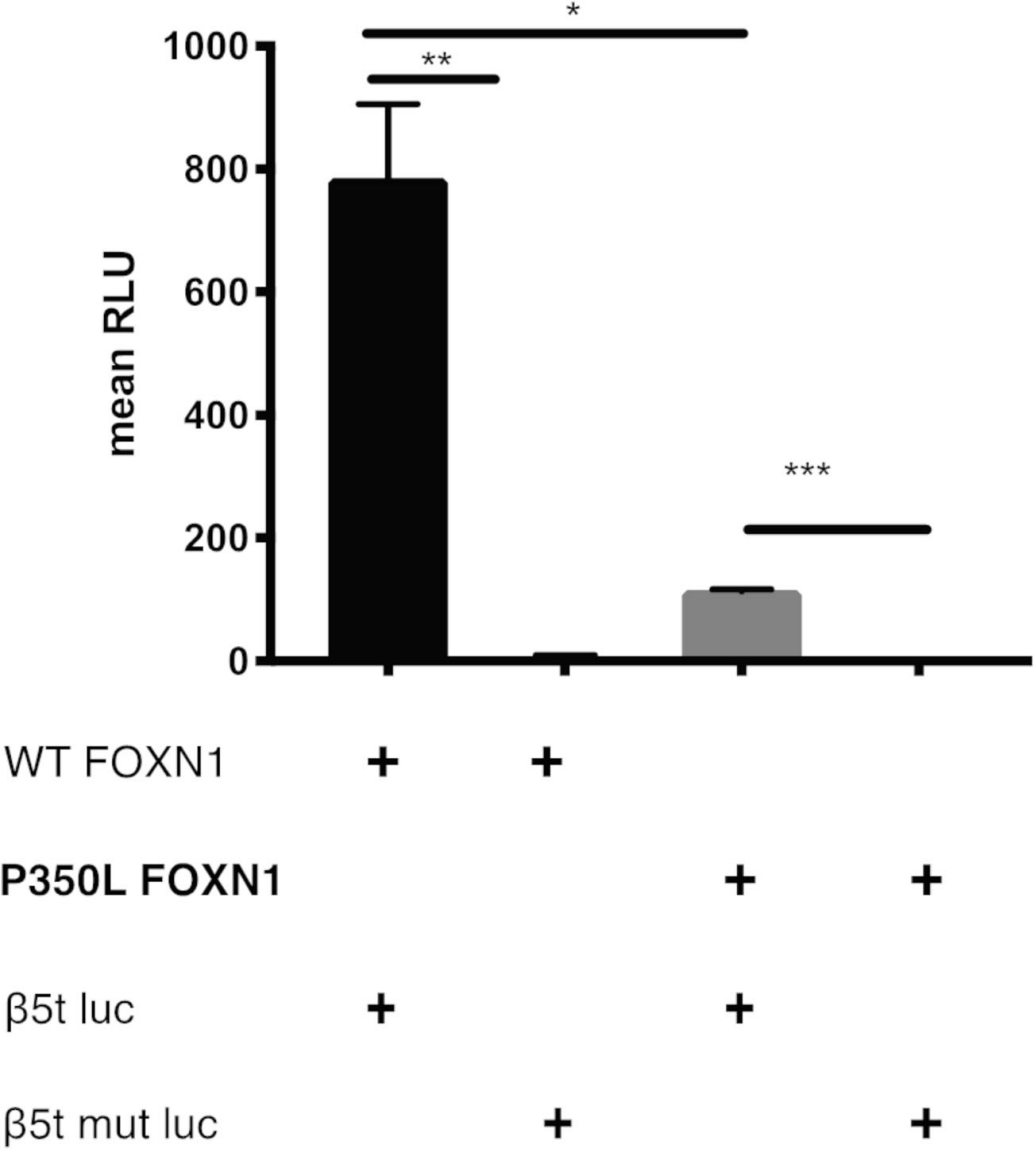
Assessing the pathogenicity of the P350L *FOXN1* variant found in P9. Luciferase reporter assay in 4D6 human cells transfected with either wild-type *FOXN1* (WT) or P350L *FOXN1* along with β5t-luc or β5t-mut-luc luciferase plasmids. RLU, relative light units; *p* values, calculated with unpaired *t* tests, *p = .0106, **p = .0080,***p = .0003

P9 was well until the age of 1.5 years when he developed generalized lymphadenopathy associated with a high EBV circulating load in peripheral blood. He did not experience any adverse reaction to the administration of live vaccines (MMR and BCG) and he did not develop any complication after varicella infection. At the age of 3 years, he was started on intravenous immunoglobulin replacement because of recurrent sinusitis associated with hypogammaglobulinemia. At the age of 6 years, he developed an EBV-related non-Hodgkin abdominal Burkitt lymphoma, successfully treated with chemotherapy. During chemotherapy, he developed hemorrhagic enteropathy and pseudomonas sepsis. At the age of 8 years, he developed Evans syndrome (Coombs-positive autoimmune hemolytic anemia and immune-mediated thrombocytopenia), persistent splenomegaly, and EBV-driven infiltrative lung disease. He was treated with rituximab, which cleared the EBV infection leading to a marked improvement of the lung CT scan and stabilization of the hemoglobin levels. Moreover, he was started on cotrimoxazole for prophylaxis against *Pneumocystis jiroveci* pneumonia (PCP). NGS revealed two novel compound heterozygous variants in *FOXN1*. The first is a nonsense mutation leading to an early stop codon and the second is a missense variant. The second variant was studied in vitro and was found to be hypomorphic (Fig. 4). Interestingly, P14, who carried a heterozygous mutation, developed an EBV-related diffuse large B cell lymphoma. Furthermore, EBV-related high-grade B cell lymphoma was the presenting manifestation in P11, at the age of 4 months.

Autoimmune manifestations were detected in P16 and P17 and included psoriasis and type 1 diabetes mellitus. While P16 also experienced severe infections during the first 2 years of life, autoimmune manifestations represented the only clinical features in P17.

### Immunological Features

**Fig. 5:**
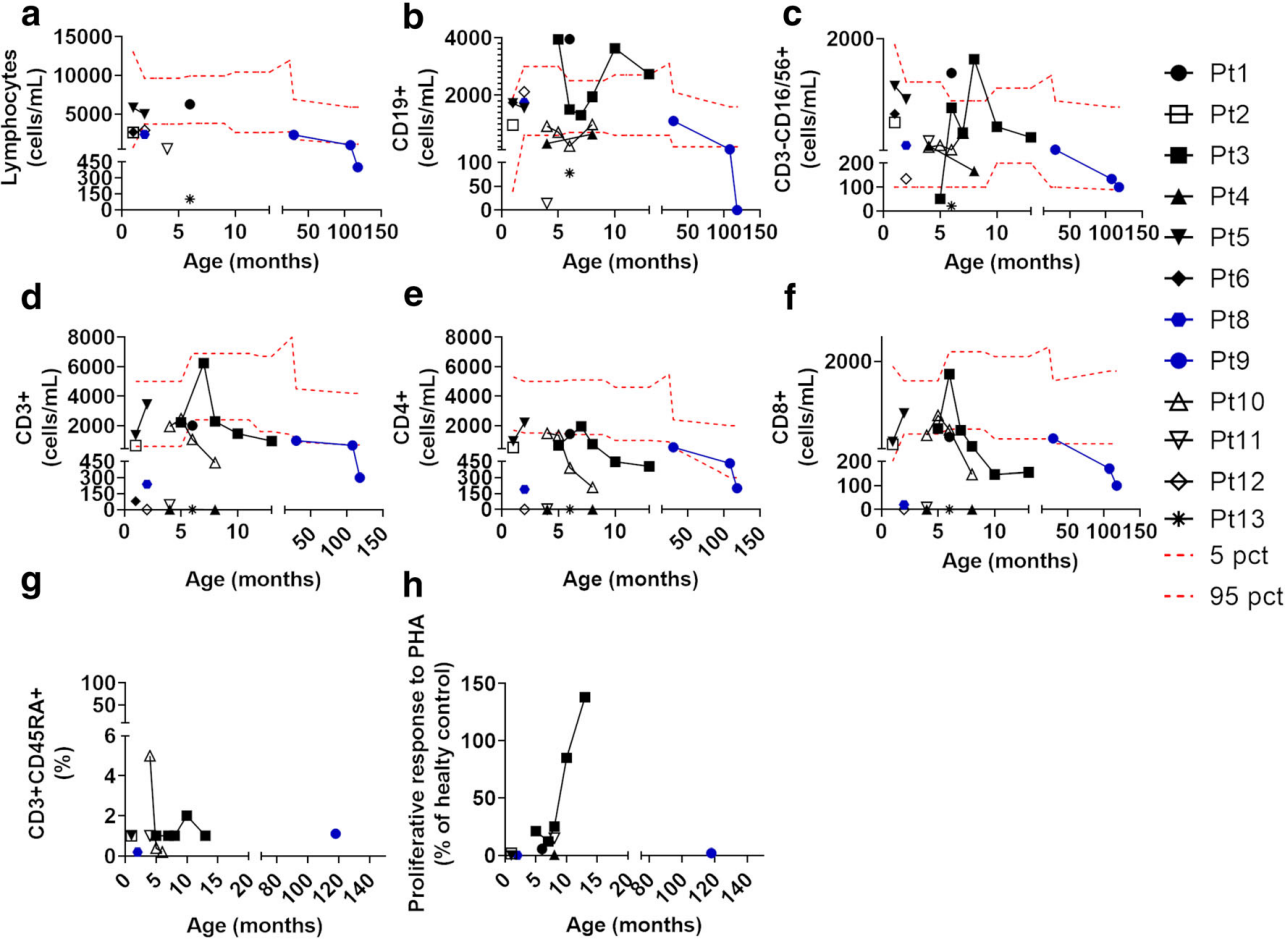
Lymphocyte subpopulations at different ages. **a** Lymphocytes. **b** B cells (CD19+). **c** NK cells (CD16/CD56+). **d** T cells (CD3+). **e** T helper (CD3+CD4+). **f** Cytotoxic T cells (CD3+CD8+). **g** Naïve T helper (CD3+CD4+CD45RA+). **h** Proliferative response to PHA expressed as % of the healthy control. In all the panels, each line and symbol represents a single patient. Blue symbols represent compound heterozygous patients. The red dashed lines represent the 5th and 95th centile levels of the age-matched reference values

The study of the T cell compartment revealed a complete absence or a reduction of T cells in all the homozygous/compound heterozygous patients (Fig. 5). In 3 patients (P3, P5, and P10), the T cell levels ranged between low and normal values (Fig. 5). It should be noted that the 5 patients showing the highest T cell levels (P1, P2, P3, P5, and P10) had OS, suggesting that, in these patients, the T cell defect may have been masked by the oligoclonal expansion of the few available T cell clones. In support of this hypothesis, there is evidence that the TCRVβ repertoire in P3, P8, P9, and P10 was markedly oligoclonal [11]. Moreover, in P3, P5, and P10, T cells had a senescent phenotype (> 95% of the T cells showed a CD45RO+ memory phenotype, and naïve and CD4+CD31+CD45RA+ RTE were undetectable in P2, P3, and P5, and were very low in P10). Maternal engraftment was excluded in P1, P3, and P10 and it was not evaluated in P2 and P5. Both CD4 and CD8 compartments were affected (Fig. 5). Naïve T cells and RTE were markedly reduced in all the patients evaluated (P2, P3, P5, P8–11) (Fig. 5). Moreover, TRECs were very low in P3, P5, P8, P9, P12, and P13. Markers of thymic output were not evaluated in P4 due to lack of T cells before transplantation and were not available for P1, P6, and P7. Moreover, in P3 and P10, a marked expansion of the αβ-double-negative T cell compartment was observed [9], while it was normal in P9. P3 also showed a marked expansion of the FoxP3+ T cell compartment. Unfortunately, these populations were not evaluated in any of the other patients.

Proliferative response to PHA and anti-CD3 was low to absent in 8 studied patients (P1, P2, P3, P4, P5, P8, P10, P11, and P13) (Fig. 5). In P9, the proliferative response was impaired when evaluated on total cells while it was normal when evaluated on separated T cells. Proliferative response was not evaluated in the remaining patients. Surprisingly, in P3, the response to PHA increased to normal values before the thymus transplant (Fig. 5) [11]. The proliferative response to PMA and ionomycin was normal in the 2 patients evaluated (P1 and P2). P5 had severe deficiency (< 1%) of switched and unswitched memory B cells, presumably as a consequence of the lack of the T helper cells. Moreover, P13 showed a severe lymphopenia resulting in a T-B-NK-SCID phenotype. Immunoglobulin levels were markedly decreased in all the patients evaluated. Specific antibody response was impaired in the 2 patients evaluated (P1 and P3). Eosinophil levels were increased in P1, P3, and P8, showing signs of OS. Similarly, IgE levels were markedly increased in P5.

P9, who was not treated until the age of 11 years, showed a progressive worsening of the T cell lymphopenia associated with absent RTE (0.5%) and very low TRECs. The patient also developed B cell lymphopenia as a result of rituximab treatment.

The 3 heterozygous siblings (P14, P15, and P18) showed a moderate lymphopenia, while lymphocyte count was normal in the 2 adult heterozygous patients (P16 and P17) (Fig. 3b). CD4 and CD8 levels were markedly reduced in the first few months of life in the 3 siblings, and they improved with age in P14 and in P15 (Fig. 3f–g). In P16 and P17, CD4 and CD8 levels were in the normal range (Fig. 3f–g). RTE and naïve CD4 and CD8 T cells were markedly reduced in all the heterozygous patients, independently of the age (Fig. 3h). Proliferative response to PHA was impaired in P14 and normal in P16 and P17. In P15 and P18, it was not evaluated. B and NK cell levels were normal in all the heterozygous patients. Immunoglobulin levels were markedly reduced in P14 and P18, who required replacement therapy, while they were normal in P16 and P17.

### Treatment and Outcome

Four homozygous/compound heterozygous patients did not receive any definitive treatment. As expected, all died within the first year of life. Interestingly, P9, who presented with late-onset atypical clinical phenotype, did not receive any definitive treatment until the age of 11 years. Four homozygous/compound heterozygous patients received hematopoietic stem cell transplant (HSCT) and 5 received thymus transplant. The survival associated with HSCT was 2 out of 4 (50%). The source of HSC was in 2 patients a matched sibling donor, in 1 patient cord blood, and in 1 patient HLA-matched unrelated donor. The source in the 2 patients who survived was a matched sibling donor in one patient and an HLA-matched unrelated donor in the other. The survival associated with thymus transplant was 4 out of 5 (80%).

Among the heterozygous patients, P15 underwent HSCT, from cord blood, and died 2 years after because of severe graft versus host disease. P14 and P16, who developed severe infections in the first years of life, showed an improvement of the phenotype in the following years and they are alive at the age of 11 and 38 years, respectively. P14 still shows persistent hypogammaglobulinemia for which she requires replacement therapy. P18 is currently 4 months old and is under intravenous immunoglobulins, cotrimoxazole and acyclovir prophylaxis.

## Discussion

In this paper, we described the expanding clinical features of the nude SCID/CID phenotype associated with *FOXN1* mutations. The cohort included 11 patients with homozygous, 2 with compound heterozygous, and 5 with heterozygous mutations.

Signs of SCID were present at onset in all homozygous, in 1 compound heterozygous, and in 4 heterozygous patients. Lung infection was the most common infectious manifestation, observed in 12/18 patients. Half of the patients presented with signs of OS. The study of lymphocyte subpopulations revealed a T-B+NK+ phenotype in most of the cases. In a few cases, T cell levels were low-to-normal. As expected, OS was associated with higher CD3 levels that were sometimes normal. However, naïve T cells and RTEs and TRECs were very low or absent in all cases tested. Low B cell levels were detected in one patient and in a second patient marked lymphopenia resulted in T-B-NK-phenotype.

In homozygous patients, alopecia and nail dystrophy, which are usually considered the hallmarks of the syndrome, were not present in all the subjects, even though nail dystrophy may be difficult to identify. In a recent paper, Du et al. described 2 patients with *FOXN1* compound heterozygous mutations who only had selective thymic hypoplasia [18]. By reproducing the mutations in mouse models generated through CRISPR-Cas9 technology, the authors confirmed that different mutations may play distinct roles in TECs and keratinocytes. In particular, they identified a region at the COOH-terminal end of the DNA binding domain that uncouples TEC development and keratinocyte differentiation [18]. In our cohort, 2 homozygous patients did not show alopecia at birth. In both patients, the mutations (S188fs and V294I) were located in the forkhead domain. However, in contrast to the 2 patients described by Du et al., in our study, the first patient developed alopecia during the follow-up in association with erythroderma and the second one had a sibling with alopecia, carrying the same mutation. In a third patient born with congenital alopecia, spontaneous hair regrowth was observed starting from 3 years of age. This patient carries 2 compound heterozygous mutations, namely a stop codon mutation (C82X) in the N-terminal domain and a missense mutation (P350L) in the forkhead domain. Alopecia was absent in 1 out of 5 members of the heterozygous family and only involved eyebrows in the remaining 4 heterozygous patients. Notably, the mutation identified in this family is very close to the mutation reported by Du et al. in the transactivation (TA) domain [18]. These observations suggest that FOXN1 activity in keratinocytes might be only partially impaired in some cases and other still unidentified factors might also play a role in regulating its activity. Alternatively, a differential dose dependency in TEC and keratinocytes with respect to FOXN1 activity may explain why certain mutations only affect TEC development. The absence of the classical hallmarks of the disease associated with the finding of normal or only slightly reduced lymphocyte count may delay the diagnosis, especially in a non-specialist setting, where the naïve compartment is not routinely assessed. Increased IgE levels and eosinophilia, also typically observed in OS, in association with erythroderma and chronic diarrhea, may also be misleading and delay the diagnosis, as observed in P6 [13]. These observations further support the importance of NBS in the timely identification of the different forms of SCID.

Thanks to the recent introduction of the NBS in combination with NGS it has been possible to unravel novel important evidence on the role of *FOXN1* in human genetics. In a recent paper, we described a cohort of 21 children, identified through NBS in which NGS led to the identification of heterozygous pathogenic variants in the *FOXN1* gene [17, 24, 25]. Most of the children showed nail dystrophy and some of them also experienced recurrent upper respiratory infections. Respiratory infections were rarer in adult heterozygous patients, who also showed normalization of CD4^+^ T cell levels. In a mouse model heterozygous for *Foxn1* mutation, we also showed that Foxn1 plays a key role in the recruitment of the early thymic precursors and that this process is also affected by *Foxn1* haploinsufficiency. In two brothers, carrying a heterozygous deletion in the C-terminal domain (P465Rfs*82) leading to a premature stop codon, the clinical phenotype was more severe, characterized by recurrent opportunistic infections and required HSCT in one patient [17]. The clinical phenotype was more similar to that observed in patients carrying homozygous mutations. These 2 patients and their younger sibling were better analyzed in our study and the phenotype was additionally evaluated in other family members carrying the same *FOXN1* variant. In particular, the father experienced severe infections requiring hospitalization and admission to the intensive care unit in the first few years of life. Differently, the paternal grandmother, who also carried the mutation, never experienced severe infections but developed type 1 diabetes mellitus and psoriasis. The three of them had eyebrow alopecia. Interestingly, the mutation identified is very close to the mutation reported by Du et al. in the TA domain [18]. In the patient described by Du et al., the mutation in the TA domain was associated with a polymorphism with no functional relevance on the other allele, suggesting that this patient was also heterozygous [18]. Even if we were not able to characterize in vitro the variant identified, the severity of the clinical phenotype suggests that FOXN1 function was more severely impaired in these patients compared to the other heterozygous subjects. Different mechanisms may explain this finding. This mutation may potentially lead to a mechanism of negative dominance. However, in our report, the clinical phenotype in the grandmother, carrying the same mutation identified in the 3 siblings, was not as severe. The observation that all the children within this family were symptomatic, while the adults were not, indicates that the requirement of FOXN1 varies with the age, as already described in heterozygous mice, whose immunological alterations improve during adulthood. Another explanation may be the presence of additional mutations in noncoding regions of *FOXN1*. Recently, Larsen et al. identified intronic regulatory elements that play a key role in tissue-specific expression of *Foxn1* in TECs [26]. In particular, the authors identified a highly conserved 1.6-kb region in the first intron of *Foxn1* whose deletion leads to a complete abrogation of Foxn1 expression and thymus development. Of note, keratinocyte differentiation is not affected by deleting this region [26]. More studies will be necessary to confirm these hypotheses. Interestingly, one of the 2 brothers developed EBV-related diffuse large B cell lymphoma, and EBV-related B cell lymphoma was the presenting manifestation in P9 and P11. EBV-related B cell lymphomas represent a well-known complication in secondary immunodeficiencies and IEIs [27]. Different IEIs affecting NK and T cell function are associated with the development of chronic EBV infections, eventually leading to lymphoma [28, 29]. In this study, 3 patients, carrying homozygous, compound heterozygous, or heterozygous mutations developed EBV-related lymphomas, suggesting that T cell lymphopenia in FOXN1-deficient patients impairs the immunological control of EBV infection. In P9, uncontrolled EBV infection also led to the development of infiltrative lung disease. P9, carrying two compound heterozygous mutations, did not show any signs of SCID at the onset. His phenotype was characterized by late-onset chronic EBV infection, leading to the development of B cell lymphoma and lung infiltration. It should be noted that in this patient, a stop mutation was associated with a missense mutation, differently from P8, presenting with a typical Nude SCID phenotype, and carrying 2 different stop mutations.

A spectrum of clinical manifestations may be associated with different mutations, and the severity of the clinical phenotype likely depends on the amount of residual activity of the gene product, as previously observed for other SCID-related genes [30]. Similarly to what recently observed by Du et al. in a patient carrying a heterozygous mutation affecting the TA domain [18], in a family carrying a heterozygous mutation, we observed an unexpectedly severe phenotype, characterized by CID in 4 out of 5 patients, suggesting that heterozygous mutations may also present with severe clinical phenotypes. A negative dominant mechanism or the presence of additional alterations in noncoding regions may explain the different severity compared to other heterozygous mutations. More studies are necessary to better characterize the effect of the different mutations and to define a clear-cut clinical strategy for delineating genotype-phenotype relationships to guide clinicians towards the best therapeutic choice. Moreover, as for other types of atypical combined immunodeficiencies, further studies are necessary to define the best therapeutic approach. In fact, both thymus transplant and HSCT may result in the development of severe complications [31–34]. Though numbers are too small to make a definitive conclusion, the survival after thymus transplantation was better than after HSCT and in three patients, there was good evidence of naïve T cell production. As already reported, homozygous mutation leads to a SCID phenotype that may befit thymus transplantation since HSCT may be ineffective. As for compound heterozygous mutations, although a milder phenotype has been reported, symptoms may be severe as well and must also be considered the risk of lymphoma. Thus, a decision on the therapeutic strategy should be taken on an individual case basis. Differently, the phenotype associated with heterozygous mutations is usually absent or very mild and no therapeutic intervention is required. Our recent study suggests that lymphopenia and susceptibility to infections in heterozygous patients tends to improve with age. This improvement is not dependent on a recovery of the thymus function and the defect of the CD8 compartment usually persists also in the adults [17]. Studies are needed to define whether the phenotype in atypical heterozygous or compound heterozygous tends to improve with age and which treatment is the most indicated in these cases.
